# The burden of suspected strokes in uMgungundlovu – Can biomarkers aid prognostication?

**DOI:** 10.4102/hsag.v28i0.1916

**Published:** 2023-05-09

**Authors:** Juan M. Jansen van Vuuren, Somasundram Pillay, Ansuya Naidoo

**Affiliations:** 1 School of Clinical Medicine, Faculty of Health Sciences, University of KwaZulu-Natal, Durban, South Africa; 2 Department of Neurology, Internal Medicine, Grey’s Hospital, Pietermaritzburg, South Africa; 3 Department of Internal Medicine, King Edward VIII Hospital, Durban, South Africa

**Keywords:** stroke, cerebrovascular accident, ischaemic stroke, haemorrhagic stroke, prognosis, prognostication, morbidity, mortality, biomarkers, developing countries, South Africa

## Abstract

**Background:**

The burden of stroke is increasing worldwide. The hierarchical healthcare referral system in South Africa (SA) poses unique challenges to clinicians when caring for people with suspected strokes (PsS). To improve health outcomes, novel strategies are required to provide adequate care, including prognostication, in SA.

**Aim:**

To determine the subjective burden of and challenges posed by suspected stroke cases and the potential usefulness of biomarkers in prognostication.

**Setting:**

This study was conducted in the uMgungundlovu Health District (UHD), KwaZulu-Natal, SA.

**Methods:**

An online questionnaire was distributed to doctors within the UHD. Demographic data and answers to a series of 5-point-Likert-type statements were collected.

**Results:**

Seventy-seven responses were analysed. A third of doctors worked in primary healthcare facilities (PHCare) and saw ≥ 2.15 suspected strokes-per-doctor-per-week, compared to ≥ 1.38 seen by doctors working in higher levels of healthcare. Neuroimaging was relied upon by > 85% of doctors, with nearly half of PHCare doctors having to refer patients to facilities 5 km – 20 km away, with resultant delays. Knowledge about prognostic biomarkers in strokes was poor, yet most doctors believed that a biomarker would assist in the prognostication process and they would use it routinely.

**Conclusion:**

Doctors in this study faced a significant burden of strokes and rely on neuroimaging to guide their management; however, many challenges exist in obtaining such imaging, especially in the PHCare setting. The need for prognostic biomarkers was clear.

**Contribution:**

This research lays the platform for further studies to investigate prognostic biomarkers in stroke in our clinical setting.

## Introduction

The global burden of cerebrovascular disease is increasing steadily and remains one of the most important causes of morbidity and mortality (Virani et al. 2020). Low-to-middle income countries (LMIC), such as South Africa (SA), are disproportionately affected when compared to wealthier nations (The World Bank 2020; Yan et al. 2016). The standard incidence of first-ever stroke was found to be highest in Tianjin, China (more than 300 per 100 000 per year) and lowest in Perth, Australia (approximately 50 per 100 000 per year) based on a review by Bejot, Daubail and Giroud (2016b). The incidence of stroke at the turn of the century had a range of 95 to 290 per 100 000 per year in Europe (Bejot et al. 2016a). A systematic review by Owolabi and colleagues revealed a pooled incidence of stroke between 25 and 250 per 100 000 person-years (Owolabi et al. 2018). Although their data included statistics from LMIC, African countries were underrepresented. This is an important caveat as LMIC in Africa represents a disproportionately high rate of poor functional outcomes (Johnston, Mendis & Mathers 2009; Kim & Johnston 2011).

Although complex, the reasons for this disparity have been well documented, and the so-called ‘epidemiological shift’ has been suggested as a possible cause (Omran 2005). As socioeconomic circumstances improve in previously impoverished communities, the burden of disease shifts from communicable to predominantly that of non-communicable diseases, a concept proven in China (Jiang et al. 2016). This transition does not occur rapidly in many communities, notably in Africa, leading to the so-called double burden of disease (Kabudula et al. 2017; Kuate Defo 2014). Sub-Saharan Africa bears the brunt of this phenomenon, where communicable and non-communicable diseases affect the lives of millions (Kuate Defo 2014; National Research Council 2012). As a result, strategies to ensure adequate care for various disorders, including stroke, need to be pursued to deliver better health outcomes.

The uMgungundlovu Health District covers approximately 9200 km^2^ of land, servicing 10% of the population of KwaZulu-Natal (KZN), where more than 85% of people do not have medical insurance and rely heavily on public health facilities (KwaZulu-Natal Department of Health 2021). The public healthcare system in SA generally follows a hierarchical referral system (Mojaki et al. 2011). Level 1 facilities or primary healthcare facilities (PHCare) include primary healthcare clinics, community healthcare centres and district hospitals, with level 2 facilities consisting of regional hospitals and level 3 facilities consisting of tertiary hospitals and specialised hospitals (KwaZulu-Natal Department of Health 2014). Levels 2 and 3 referral facilities are generally known as the higher level of healthcare facilities (HLHCare). In SA, most people with suspected stroke (PsS) present to PHCare, where access to advanced investigations, such as neuroimaging, are not readily available (Taylor & Ntusi 2019). Referral of PsS from PHCare to HLHCare is indicated in certain cases, especially when emergency neuroimaging, notably computed tomography scans (CT), are required (Bryer et al. 2011). Understanding the burden of disease (in the form of patient load), including strokes, presenting to different levels of healthcare, has implications for resource allocation (Kluge 2007).

Early identification, confirmation and management of suspected strokes, which includes prognostication, improve clinical outcomes (Marler et al. 2000; Phipps & Cronin 2020). Neuroimaging in the form of an uncontrasted CT of the brain is the gold standard in the diagnosis of a stroke and plays an important role in prognostication; however, it is not readily available to all patients presenting with suspected strokes (Piliszek et al. 2016). A high index of suspicion is therefore required by clinicians based on potential clinical presentations of PsS. These presentations vary based on the location of the intracranial insult (Murphy & Werring 2020; Tadi & Lui 2022). The well-known acronym FAST (facial weakness, arm weakness, slurred speech, time) is widely utilised to identify early features of suspected strokes by clinicians (Pickham et al. 2019).

Research has revealed the potential value of additive predictive models, such as biomarkers, in determining prognosis following stroke (Bustamante et al. 2016). More than 58 biomarkers used in the various stages of diagnosis, management, monitoring and prognostication of strokes have been identified (Dolmans et al. 2019; Donkel et al. 2019; Jickling & Sharp 2015; Martin & Price 2018; Misra et al. 2017, 2020; Whiteley et al. 2008). The use of biomarkers to assist in prognostication following a stroke has been discussed in a recently published scoping review (Jansen Van Vuuren et al. 2022). Many of the available biomarkers are not readily available in SA either because of prohibitive cost or the requirement of specialised equipment to perform the laboratory investigations. The potential benefits of such biomarkers have not yet been explored within our setting.

Data on the subjective burden of suspected strokes at different levels of healthcare facilities are not readily available. In addition to quantifying this subjective burden, this study aimed to highlight and compare some of the challenges posed by suspected stroke cases and resources available to doctors working within different levels of healthcare, who care for patients with suspected strokes. In addition, this study aimed to assess their perspectives on the potential value of a prognostic biomarker in the setting of suspected strokes.

## Methods

### Study design

The cross-sectional survey aimed:

- To determine the subjective burden of strokes experienced by doctors at different levels of healthcare.
- To highlight and compare some of the challenges posed by suspected stroke cases within different levels of healthcare.
- To determine the perspectives of doctors on the potential value of prognostic biomarkers in the setting of suspected strokes.

### Study setting

uMgungundlovu Health District (UHD) public healthcare facilities (including primary healthcare clinics, community health centres, district hospitals, regional hospitals, tertiary hospitals and specialised hospitals), KZN, SA. These facilities serve a population living in rural, peri-urban and urban settings and include people of numerous diverse ethnic backgrounds.

For the purposes of analyses primary healthcare clinics, community healthcare centres and district hospitals were grouped under the term PHCare settings, while regional hospitals, tertiary hospitals and specialised hospitals were grouped as HLHCare settings.

### Population

The study population consisted of medical doctors (including medical interns, community service medical officers, medical officers, registrars and consultants) working within the public sector in the UHD.

Medical interns and community service medical officers were grouped under the term junior doctor, medical officers and registrars of all grades were grouped as middle-grade doctors and consultants comprised their own group.

### Sampling method and sample size

Non-probabilistic, purposive, directed sampling was used. This method was utilised to allow for maximum information extraction within a limited population. The required sample size is not easily calculable because of the heterogeneity of staffing within various facilities throughout the UHD, and the exact number of doctors working within the UHD is not known.

Therefore, the sample size has been calculated based on the assumption that the UHD has an average number of doctors equal to the national average, that is, 0.79 doctors per 1000 population. The UHD serves a population of 1 052 730 people. This means the anticipated number of doctors is approximately 832 (rounded up to the nearest whole number). The required sample size was 41, with a confidence interval of 95% and a margin of error of 15% (because of the uncertainties described above).

### Data collection

Data were collected utilising an online Google^®^ Forms questionnaire. This resource was decided upon based on its ease of access, ease of use and security features. Data are stored on a password-secured computer, with access available only to the authors. The link to the online questionnaire was distributed among doctors within the UHD by word of mouth and local and institutional networking, including messaging applications and social media. Prior to entering the questionnaire, the participant was required to confirm that they were working as a doctor within the public sector of the UHD.

The data collection took place over a 1-month period (July 2021 – August 2021) and achieved a sample size of 77.

### Instrument

The questionnaire was designed following consultation with available literature, including extensive work done on a recently published review article, as well as two experts within the field of neurology and piloted on a very small sample (3) to ensure scientific rigour and ease of operation. The validity and reliability of this instrument have not previously been tested, and this is the first study to address the specific aims set out.

The questionnaire assessed a number of variables.

Basic demographic data were collected:

- The level of care in which the participants were working (primary healthcare clinics, community health centres, district hospitals, regional hospitals, tertiary hospitals and specialised hospitals).
- The level of practice of the participants (medical interns, community service medical officers, medical officers, registrars and consultants).
- The discipline in which the participants worked (primary care, family medicine, emergency medicine, internal medicine, neurology or other).

The participants were asked to quantify the number of PsS seen per week (0, 1–3, 4–6, 7–10 or more than 10) to ascertain the subjective burden of stroke.

To highlight challenges faced by doctors at various levels of healthcare, data pertaining to access to neuroradiological imaging services were collected:

- Access to on-site CT (yes, no or during working hours only)
- The distance (N/A – on-site access, 0 km – 5 km, 5 km – 20 km, more than 20 km)
- The time to neuroimaging (0–30 min, 30 min – 1 h, 1h – 4 h, 4h – 12 h, more than 12 h).

To highlight challenges faced by doctors at various levels of healthcare, data pertaining to access laboratory services were collected:

- Access to on-site laboratory (yes or no).

Lastly, a series of 5-point, ordinal Likert-type statements were posed to the participants (where 1 represents ‘Strongly disagree’, 2 ‘Disagree’, 3 ‘Neutral’, 4 ‘Agree’ and 5 represents ‘Strongly agree’). The following statements were assessed:

- Neuro-imaging plays an important role in determining my management plan in patients with suspected stroke (to determine current practices).
- I feel I have enough knowledge with regard to the use of biomarkers in prognosticating patients with stroke (to determine knowledge on the topic).
- A readily available biomarker that could aid in prognostication of patients with suspected stroke would be valuable in my setting (to determine beliefs).
- If such a biomarker existed, I would perform the test routinely in my patients with suspected stroke (to determine attitudes towards the topic).

For some analyses, answers were dichotomised to agree (Likert items agree and strongly agree) and disagree (Likert items disagree and strongly disagree). This was done in accordance with other literature to allow for ease of specific interpretation at the cost of some statistical power.

### Statistical analysis

Simple descriptive analyses have been performed, with the results discussed below. To test whether significant differences in answers arose when comparing different levels of healthcare, the non-parametric, categorical variables were assessed using the Fisher’s exact test (because of the small sample size). Statistical significance was placed at a *p* value of < 0.05, with a confidence interval of 95% and a margin of error of 5%. SigmaXL version 9.03 MAC was utilised for all statistical analyses. All values were rounded to the first decimal place. Percentages do not always add up to 100% because of rounding.

### Ethical considerations

Informed consent was obtained from all participants. The participants were presented with an information sheet and consent form upon accessing the online questionnaire and only if they agreed and/or consented, were they able to proceed with the questionnaire. This study was approved by the Biomedical Research Ethics Committee (BREC) of the University of KwaZulu-Natal (UKZN) (BREC reference number: BREC/00002521/2021) on 14 June 2021.

## Results

A total of 81 respondents consented to participate in the questionnaire. Following the exclusion of four respondents who were not currently working as doctors within the UHD a total of 77 responses were included for analyses. The demographic data are represented in Table 1. Most doctors worked within HLHCare were middle-grade doctors. There was a significant difference in the levels of practice between PHCare and HLHCare, with the majority of the respondents from the former being staffed by junior doctors when compared to the latter (*p* < 0.01). The majority of doctors who responded to the survey work within internal medicine and family medicine.

**TABLE 1:** Demographic data.

Demographic	*n*	% of Total
**Facility level of care: PHCare**		
Primary healthcare clinic	6	7.8
Community healthcare centre	2	2.6
District hospital	19	24.7
Sub-total	27	35.1
**Facility level of care: HLHCare**		
Regional hospital	18	23.4
Tertiary hospital	30	39.0
Specialised hospital	2	2.6
Sub-total	50	65.0
Grand Total	77	100
**Level of practice: Junior doctors**		
Medical intern	15	19.5
Community service medical officer	6	7.8
Total	21	27.3
**Level of practice: Middle grade doctors**		
Medical officer, grade 1	21	27.3
Medical officer, grade 2	9	11.7
Medical officer, grade 3	3	3.9
Registrar	10	13.0
Total	43	55.9
Consultant	13	16.9
**Discipline**		
Primary care	8	10.4
Family medicine	17	22.1
Emergency medicine	8	10.4
Internal medicine	21	27.3
Neurology	4	5.2
Other	19	24.7

PHC, primary healthcare facilities; HLHC, higher level of healthcare facilities.

**FIGURE 1:**
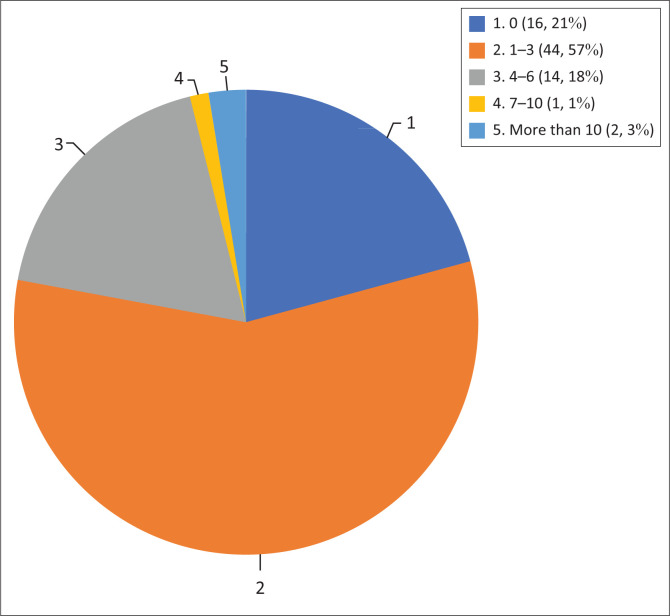
Average number of strokes seen per doctor, per week.

The subjective burden of strokes as reported by the doctors are summarised in Figure 1. Nearly 60% of doctors consult between 1 and 3 suspected strokes per week. Using the lower limit of the proposed ranges of suspected stroke cases seen per week, doctors working in PHCare see a total of ≥ 58 stroke cases per week compared to colleagues working in HLHCare, who see a total of ≥ 69 stroke cases per week. This is a mean of ≥ 2.15 strokes per doctor in PHCare per week and ≥ 1.38 strokes per doctor in HLHCare per week.

Table 2 summarises the access and distance from the doctor’s healthcare facility to the nearest facility with neuroradiological services, as well as the time from when a patient presents to the doctor with a suspected stroke to obtaining neuroimaging. None of the participants working in PHCare had on-site access to neuroimaging, while 90% of those working in HLHCare had on-site access. Nearly half of doctors without on-site neuroradiological facilities reported that their nearest referral facility with neuroimaging capabilities is 5 km – 20 km away. The delay between patient presentation to neuroimaging differs greatly between PHCare and HLHCare, with the former having much longer delays when compared to the latter. When comparing the time from presentation to neuroimaging (dichotomised to < 4 h and > 4 h) between PHCare and HLHCare, a statistically significant difference is evident (*p* < 0.01).

**TABLE 2:** Access, distance and time to neuroimaging.

Variable	PHCare		HLHCare	
	*n*	%	*n*	%
**On-site CT**				
Yes	0	0	45	90
No	27	100	2	4
During working hours only	0	0	3	6
**Distance to nearest CT capable facility**				
N/A – on site access	0	0	48	96
0 km – 5 km	10	37.0	2	4
5 km – 20 km	13	48.2	0	0
More than 20 km	4	14.8	0	0
**Time to CT**				
0 min – 30 min	1	3.7	3	6
30 min – 1 h	0	0	5	10
1 h – 4 h	3	11.1	28	56
4 h – 12 h	6	22.2	10	20
More than 12 h	17	63.0	4	8

PHC, primary healthcare facilities; HLHC, higher level of healthcare facilities; CT, computed tomography; N/A, not applicable.

On-site laboratory services were available to the vast majority (68; 88.3%) of participants. The 8 (10.4%) doctors who did not have access were primarily from PHCare with only 1 (1.3%) doctor from a specialised hospital reporting not having access.

Responses to the statement ‘Neuro-imaging plays an important role in determining my management plan in patients with suspected stroke’ are summarised in Table 3. The median response was 5 (‘Strongly Agree’; Interquartile range [IQR] 4–5). Answers did not differ based on participant characteristics, including levels of practice, burden of strokes, distance or time to neuroradiological services. When the answers were dichotomised, it is evident that the majority of doctors (66% or 85.7%) rely on neuroimaging to guide management of PsS. The Fisher’s exact test revealed no difference when comparing the dichotomised reliance on neuroimaging when comparing responses from PHCare and responses from HLHCare (*p* = 0.2).

**TABLE 3:** Likert-type answers in relation to participant characteristics to the statement: Neuro-imaging plays an important role in determining my management plan in patients with suspected stroke.

Variable	Strongly disagree		Disagree		Neutral		Agree		Strongly agree	

	*n*	%	*n*	%	*n*	%	*n*	%	*n*	%
Overall	1	1.3	2	2.6	8	10.4	20	26.0	46	59.7
**Levels of care**										
PHCare	1	3.7	1	3.7	6	22.2	5	18.5	14	51.9
HLHCare	0	0	1	2.0	2	4.0	15	30.0	32	64.0
**Levels of practice**										
Junior doctors	0	0	2	9.5	5	23.8	5	23.8	9	42.9
Middle-grade doctors	1	2.3	0	0	2	4.7	11	25.6	29	67.4
Consultants	0	0	0	0	1	7.7	4	30.8	8	61.5
**Burden of strokes**										
0	0	0	1	6.1	0	0	3	18.8	12	75.0
1–3	0	0	1	2.3	6	13.6	10	22.7	27	61.4
4–6	1	7.1	0	0	2	14.3	6	42.9	5	35.7
7–10	0	0	0	0	0	0	0	0	1	100
More than 10	0	0	0	0	0	0	1	50.0	1	50.0
**Distance to neuroradiology**										
On-site	0	0	1	8.3	2	16.7	2	16.7	7	58.3
0 km – 5 km	0	0	0	0	3	23.1	3	23.1	7	53.8
5 km – 20 km	1	25.0	0	0	1	25.0	1	25.0	1	25.0
More than 20 km	0	0	1	2.1	2	4.2	14	29.2	31	64.6
**Time to neuroradiology**										
0 min – 30 min	0	0	0	0	0	0	1	25.0	3	75
30 min – 1 h	0	0	1	3.2	3	9.7	9	29.0	18	58.1
1 h – 4 h	0	0	0	0	0	0	1	20.0	4	80.0
4 h –12 h	0	0	1	6.3	3	18.8	4	25.0	8	50.0
More than 12 h	1	5.0	0	0	2	10.0	5	25.0	13	65.0

PHC, primary healthcare facilities; HLHC, higher level of healthcare facilities.

Responses to the statement ‘I feel I have enough knowledge with regard to the use of biomarkers in prognosticating patients with stroke’ are summarised in Table 4. The median response was 2 (‘Disagree’; IQR 1–3). Answers did not differ based on levels of practice, burden of strokes, distance or time to neuroradiological services. When the answers were dichotomised, it is evident that the majority of doctors (51% or 66.2%) have a poor baseline knowledge of biomarkers for prognostication of PsS. Fisher’s exact test revealed no significant difference in knowledge of biomarkers when comparing the dichotomised answers of doctors from PHCare and doctors from HLHCare (*p* = 0.3).

**TABLE 4:** Likert-type answers in relation to participant characteristics to the statement: I feel I have enough knowledge with regard to the use of biomarkers in prognosticating patients with stroke.

Variable	Strongly disagree		Disagree		Neutral		Agree		Strongly agree	

	*n*	%	*n*	%	*n*	%	*n*	%	*n*	%
Overall	30	39.0	21	27.3	12	15.6	8	10.4	6	7.8
**Levels of care**										
PHCare	8	29.6	5	18.5	8	29.6	4	14.8	2	7.4
HLHCare	22	44.0	16	32.0	4	8.0	4	8.0	4	8.0
**Levels of practice**										
Junior doctors	7	33.3	5	23.8	4	19.0	4	19.0	1	4.8
Middle-grade doctors	20	46.5	11	25.6	6	14.0	3	7.0	3	7.0
Consultants	3	23.1	5	38.5	2	15.4	1	6.7	2	15.4
**Burden of strokes**										
0	8	50.0	5	31.3	1	6.3	2	1.3	0	0
1–3	17	38.6	10	22.7	10	22.7	4	9.1	3	6.8
4–6	4	28.6	4	28.6	1	7.1	2	14.3	3	21.4
7–10	1	100	0	0	0	0	0	0	0	0
More than 10	0	0	2	100	0	0	0	0	0	0
**Distance to neuroradiology**										
On-site	3	25.0	4	33.3	2	16.7	3	25.0	0	0
0 km – 5 km	4	30.8	2	15.4	4	30.8	1	7.8	2	15.4
5 km – 20 km	1	25.0	1	25.0	2	50.0	0	0	0	0
More than 20 km	22	45.8	14	29.2	4	8.3	4	8.3	4	8.3
**Time to neuroradiology**										
0 min – 30 min	0	0	1	25.0	1	25.0	1	25.0	1	25.0
30 min – 1 h	16	51.6	6	19.4	5	16.1	2	6.5	2	6.5
1 h – 4 h	0	0	4	80.0	0	0	0	0	1	20.0
4 h –12 h	6	37.5	6	37.5	0	0	3	18.8	1	6.3
More than 12 h	8	38.1	4	19.0	6	28.6	2	9.5	1	4.8

PHC, primary healthcare facilities; HLHC, higher level of healthcare facilities.

Responses to the statement ‘A readily available biomarker which could aid in prognostication of patients with suspected stroke would be valuable in my setting’ are summarised in Table 5. The median response was 5 (‘Strongly Agree’; IQR 4–5). Answers did not differ based on levels of practice, the burden of strokes, distance or time to neuroradiological services. When the answers were dichotomised, it is evident that the majority of doctors (60% or 77.9%) reported that readily available biomarker would aid in prognostication of patients following a stroke. Fisher’s exact test revealed no significant difference in answers when comparing doctors working in PHCare and those working in HLHCare (*p* = 0.7).

**TABLE 5:** Likert-type answers in relation to participant characteristics to the statement: A readily available biomarker that could aid in prognostication of patients with suspected stroke would be valuable in my setting.

Variable	Strongly disagree		Disagree		Neutral		Agree		Strongly agree	

	*n*	%	*n*	%	*n*	%	*n*	%	*n*	%
Overall	1	1.3	5	6.5	11	14.5	13	16.9	47	61.0
**Levels of care**										
PHCare	1	3.7	0	0	4	14.8	3	11.1	18	66.7
HLHCare	0	0	5	10.0	6	12.0	10	20.0	29	58.0
**Levels of practice**										
Junior doctors	1	4.8	0	0	3	14.3	4	19.0	13	61.9
Middle grade doctors	0	0	3	7.0	8	18.6	6	14.0	26	60.5
Consultants	0	0	2	15.4	0	0	3	23.1	8	51.5
**Burden of strokes**										
0	0	0	1	6.3	1	6.3	1	6.3	13	81.3
1–3	1	2.3	1	2.3	9	20.5	9	20.5	24	54.5
4–6	0	0	2	14.3	1	7.4	2	14.3	9	64.8
7–10	0	0	0	0	0	0	1	100	0	-
More than 10	0	0	1	50.0	0	0	0	0	1	60.0
**Distance to neuroradiology**										
On-site	1	8.3	1	8.3	1	8.3	0	0	9	75.0
0 km – 5 km	0	0	0	0	3	23.1	3	23.1	7	53.8
5 km – 20 km	0	0	0	0	1	25.0	0	0	3	75.0
More than 20 km	0	0	4	8.3	6	12.5	10	20.8	28	58.3
**Time to neuroradiology**										
0 min – 30 min	0	0	0	0	1	25.0	0	0	3	75.0
30 min – 1 h	0	0	4	12.9	5	16.1	4	12.9	18	58.1
1 h – 4 h	0	0	0	0	0	0	2	40.0	3	60.0
4 h –12 h	1	6.3	1	6.3	0	0	4	25.0	10	62.5
More than 12 h	0	0	0	0	5	23.8	3	14.3	13	61.9

PHC, primary healthcare facilities; HLHC, higher level of healthcare facilities.

Responses to the statement ‘If such a biomarker existed, I would perform the test routinely in my patients with suspected stroke’ are summarised in Table 6. The median response was 5 (‘Strongly Agree’; IQR 5–5). Answers did not differ based on levels of practice, burden of strokes, distance or time to neuroradiological services. When the answers were dichotomised, the vast majority of respondents (71% or 92.2%) would routinely use a biomarker to assist in the prognostication of patients following a stroke. Fisher’s exact test revealed no significant difference in answers when comparing respondents working in PHCare and those working in HLHCare (*p* = 0.7).

**TABLE 6:** Likert-type answers in relation to participant characteristics to the statement: If such a biomarker existed, I would perform the test routinely in my patients with suspected stroke.

Variable	Strongly disagree		Disagree		Neutral		Agree		Strongly agree	

	*n*	%	*n*	%	*n*	%	*n*	%	*n*	%
Overall	1	1.3	3	3.9	2	2.6	13	16.9	58	75.3
**Levels of care**										
PHCare	0	0	1	3.7	0	0	5	18.5	21	77.8
HLHCare	1	2.0	2	4.0	2	4.0	8	16.0	37	74.0
**Levels of practice**										
Junior doctors	0	0	1	4.8	0	0	7	33.3	13	61.9
Middle-grade doctors	1	2.3	1	2.3	1	2.3	3	7.0	37	86.0
Consultants	0	0	1	7.8	1	7.8	3	23.1	8	61.5
**Burden of strokes**										
0	1	6.3	0	0	0	0	1	6.3	14	87.5
1–3	0	0	2	4.5	1	2.3	10	22.7	31	70.5
4–6	0	0	0	0	1	7.1	1	7.1	12	85.7
7–10	0	0	0	0	0	0	1	100	0	-
More than 10	0	0	1	50	0	0	0	0	1	50
**Distance to neuroradiology**										
On-site	0	0	1	8.3	0	0	1	8.3	10	83.3
0 km – 5 km	0	0	0	0	0	0	4	30.8	9	69.2
5 km – 20 km	0	0	0	0	0	0	1	25.0	3	75.0
More than 20 km	1	2.1	2	41.7	2	41.7	7	14.6	36	75.0
**Time to neuroradiology**										
0 min – 30 min	0	0	0	0	0	0	1	25.0	3	75.0
30 min – 1 h	1	3.2	2	6.5	2	6.5	3	9.7	23	74.2
1 h – 4 h	0	0	0	0	0	0	0	0	5	100
4 h –12 h	0	0	0	0	0	0	5	31.3	11	68.8
More than 12 h	0	0	1	4.8	0	0	4	19.0	16	76.2

PHC, primary healthcare facilities; HLHC, higher level of healthcare facilities.

## Discussion

Stroke management, including prognostication, is evolving and requires novel strategies to ensure that the best possible outcomes are achieved for patients following a stroke. Understanding the epidemiology of a disease, including its burden, within a population served, leads to better clinical outcomes (McAllister & Wild 2009). This study highlighted the subjective burden of strokes at different levels of healthcare within the UHD.

The burden of stroke cases differed significantly between doctors working in PHCare when compared with doctors working in HLHCare, with the former seeing nearly twice as many stroke cases per week. This is in keeping with literature that shows that a significant number of individuals with suspected strokes access primary healthcare as their first port of call (Roebers et al. 2007). The Ideal Health Facility guidelines advise referral from primary healthcare clinics and community healthcare centres to district hospitals first, followed by referral to HLHCare, providing another possible explanation for these findings (South African National Department of Health 2020). The need for better resources both in the form of equipment and staff at PHCare level is evident.

Further to this, it is important to consider the workforce within the different levels of healthcare. More than 80% of respondents from PHCare work in the disciplines of family medicine and primary care. Doctors who completed the survey and were working in the PHCare setting constituted approximately one third of participants when compared to doctors working in HLHCare. This is likely because of a sampling bias produced, because the authors work in tertiary hospitals; however, appropriate statistical analyses were performed to account for this. Middle-grade doctors accounted for more than half of responses, followed by junior doctors and consultants, the latter constituting the minority. It is important to consider that doctors who participated in this survey, working in PHCare, tended to be more junior when compared to doctors working in HLHCare. This perspective is important as a study from the United Kingdom revealed that junior-grade doctors benefit from standardised tools for the management and long-term decision making in suspected stroke cases (Patel 2015). Biomarkers constitute one such ancillary tool and Boyd et al. published a consensus statement, which identified the potential use of biomarkers in stroke recovery (Boyd et al. 2017).

This study highlighted the available resources within the various levels of healthcare, as well as the challenges faced by doctors attending to PsS. Numerous studies agree that non-contrast CT scans form the cornerstone of neuroimaging evaluation to determine management strategies, including prognostication of PsS (Beauchamp et al. 1999; Latchaw et al. 2009; Packard et al. 2003). More than 85% of all doctors and more than 70% of doctors from PHCare reported that neuroimaging plays an important role in decision making, including prognostication, of PsS. The use of neuroimaging is hindered by the fact that none of the respondents from PHCare have on-site access to neuroimaging facilities. The distance to the nearest facility was mostly reported to be between 5 km and 20 km, which results in a significant delay in obtaining emergency neuroimaging. Nearly two thirds of doctors working in PHCare reported a waiting period of more than 12 h from suspected stroke presentation to obtaining neuroimaging. The implications on the decision making process, especially the eligibility for thrombolysis, are significant. The decision to manage eligible patients with thrombolysis is time sensitive and must be initiated within 3 h (or 4.5 h in select patients) from symptom onset (Powers et al. 2018). As a result, most patients presenting to PHCare would not be eligible for thrombolysis because of a lack of timeous neuroimaging, even if they were otherwise eligible, potentially worsening clinical outcomes (Messe et al. 2016). In addition, neuroimaging findings have prognostic implications, notably in the acute setting (Zhou, Zhang & Lou 2020).

In contrast to emergency neuroimaging, most doctors working in PHCare have on-site laboratory access. The role of blood biomarkers is well known and widely used in clinical practice. Cardiac markers, for example, play a key role in the diagnosis and management of suspected myocardial ischaemia (Wang et al. 2020). Numerous biomarkers have been identified for use as diagnostic and prognostic tools in the setting of stroke, with some showing more promise than others (Laskowitz et al. 1998; Makris et al. 2018; Maas & Furie 2009; Misra et al. 2020).

Doctors reported limited knowledge about biomarkers and their potential use in stroke care, and this was independent of level of care. This presents an opportunity for dissemination of information on the existence of potential biomarkers to colleagues faced with a high burden of stroke in the setting of limited resources to aid in prognostication.

More than three quarters of doctors believed that a readily available biomarker would be beneficial in the process of prognostication of patients following strokes. All but one respondent working in PHCare reported that they would use such a biomarker routinely. The tabulated results suggest that doctors working in PHCare were more likely to find the concept of such a biomarker clinically useful. This may reflect the challenges faced by doctors working in PHCare, where resources to aid in the prognostication process are not readily available.

The cost of potential biomarkers and their applicability within the South African context has been reported in a recently published scoping review (Jansen Van Vuuren et al. 2022). The cost of some of the currently available biomarkers may limit its immediate implementation. The introduction of the National Health Insurance bill, however, may provide a unique opportunity to explore and implement biomarkers previously thought to be too expensive.

This study has a number of limitations. Firstly, although the sample size exceeded the minimum required of 41, it did not meet the prerequisites for parametric data, with the result that parametric tests, which have a greater statistical power, could not be used. Secondly, sampling bias affected the ratio of participants from various clinical settings, limiting the generalisability of the findings. Attempts were made to correct for this by grouping and dichotomising where possible, at the risk of reducing the statistical power. Thirdly, Likert type questions tend to introduce central tendency bias as well as acquiescence bias, which were corrected for by introducing dichotomy and the use of directed questions.

## Conclusion

In conclusion, this study highlights the subjective burden of strokes, compared the challenges in caring for PsS between various levels of care and determined the perspectives of doctors on the potential value of prognostic biomarkers. Doctors working at all healthcare levels rely heavily on neuroimaging, but those practising in PHCare, who see the most cases, have the poorest access to neuroimaging. Doctors across all levels of care have readily accessible laboratory services and would choose to use an appropriate biomarker, if one were available, for the prognostication of stroke. The need for a readily available biomarker in the setting of stroke to aid in prognostication is therefore evident. This lays the platform for further studies to investigate potential biomarkers in our clinical setting.
